# Data on the cytotoxicity of chlorogenic acid in 3D cultures of HT-29 cells

**DOI:** 10.1016/j.dib.2023.109527

**Published:** 2023-08-28

**Authors:** M. Daniela Vélez, Johanna Pedroza-Díaz, Gloria A. Santa-González

**Affiliations:** Semillero CiBi, Grupo de Investigación e Innovación Biomédica, Facultad de Ciencias Exactas y Aplicadas, Instituto Tecnológico Metropolitano, Medellin 050034, Colombia

**Keywords:** Colorectal cancer, Phytochemicals, Chlorogenic acid, Cytotoxicity, Spheroids

## Abstract

Functional foods, beyond basic nutrition, offer health benefits to consumers thanks to the presence of bioactive compounds such as some phytochemicals [1,2]. Today, these foods are of particular interest in biomedical research due to their chemopreventive potential, as they have been shown to induce various biological effects on tumor cells, including the ability to inhibit cell proliferation, induce apoptosis, arrest cell cycle progression, and increase reactive oxygen species [3,4]. Multiple studies have confirmed the relationship between diet and the onset and progression of colorectal cancer (CRC), a malignant neoplasm that arises in the lining of the colon and/or rectum. Therefore, finding foods that can intervene in the carcinogenesis process is an important avenue of research [5,6].

Chlorogenic acid (CGA) is one of the most abundant phenolic compounds in coffee and is also found in fruits and vegetables. Scientific evidence suggests that CGA has chemopreventive potential on CRC cells [7], [8], [9]. For example, in previous studies conducted by our research group, green and roasted coffee extracts were characterized, and this compound was identified as the most abundant [7]. In addition, it was found to significantly decrease cell viability, reduce migration capacity, cause DNA fragmentation, and induce the production of reactive oxygen species in colorectal adenocarcinoma cells cultured in monolayer and treated with different doses of CGA. Furthermore, the mechanism underlying this biological activity has been related to CGA's ability to modulate the Wnt- /β-catenin pathway, which is implicated in the development and progression of CRC [7,10,11].

This paper presents data on the cytotoxic response of CGA treatments on HT-29 cells cultured in a 3D model. To this end, morphological changes in cell spheroids, propidium iodide and DiOC_6_ uptake, quantification of reactive oxygen species (ROS) production, phosphatidylserine exposure, and cell cycle progression were evaluated by flow cytometry.

**Specifications Table**

**Table:** 

Subject	Biological science
Specific subject area	Cancer research and cell biology
Type of data	Image, graph, and figure
How the data were acquired	Optical microscopy and flow cytometry (BD LSRFortessa™)
Data format	Raw Analyzed
Description of data collection	Flow cytometry was employed to quantify propidium iodide and DiOC_6_ uptake, ROS production, phosphatidylserine exposure, and cell cycle progression. In addition, cell spheroids were analyzed by light microscopy to identify morphological changes.
Data source location	Grupo de Investigación e Innovación Biomédica, Faculty of Exact and Applied Sciences, Instituto Tecnológico Metropolitano, Medellin 050034, Colombia.
Data accessibility	The raw and analyzed data files are available in Mendeley Data V2. https://data.mendeley.com/datasets/xmhtd2mtks/2[12]
Related research articles	• Villota, H.; Santa-González, G.A.; Uribe, D.; Henao, I.C.; Arroyave-Ospina, J.C.; Barrera-Causil, C.J.; Pedroza-Díaz, J. Modulatory Effect of Chlorogenic Acid and Coffee Extracts on Wnt/β-Catenin Pathway in Colorectal Cancer Cells. Nutrients 2022, 14, 4880. https://doi.org/10.3390/nu14224880 [7]

## 1 Value of the Data


 

• These data provide the first experimental evidence of the cytotoxic effect of chlorogenic acid (CGA) on HT-29 colorectal adenocarcinoma cells cultured in a 3D model.
• Previous results obtained in our laboratory allowed us to determine that CGA has promising biological activity in the context of CRC. Specifically, we reported its modulatory effect on cell proliferation, cell death, and cell migration and invasion capacity in 2D *in vitro* models. Validation of some of these findings in 3D models provides a better description of the efficacy and toxicity of CGA for CRC.
• *In vivo*, a tumor cell is surrounded by extracellular matrix and other cells that interact with each other and the environment to form a 3D complex. The results obtained in 3D cultures provide a more accurate prediction of the biological effect of CGA, so that they can potentially be used in *in vivo* models and subsequent clinical trials to validate the use of this compound in CRC.
• Drug development is a complex process that begins with the screening of ‘hits,’ or molecules with biological activity, followed by the determination of their pharmacokinetic properties. The molecules of interest, also known as lead molecules, are identified and chemically modified to enhance their pharmaceutical properties. In this context, the data reported here actively contribute to drug development and can potentially be used in later phases of this process.
• CGA is a widely distributed compound in nature, found in various plants and fruits. Establishing that it is a potential chemopreventive compound in CRC could add value to the products that contain it.

## 2 Data Description

### 2.1 Morphological Changes in Cell Spheroids

**Fig. 1:**
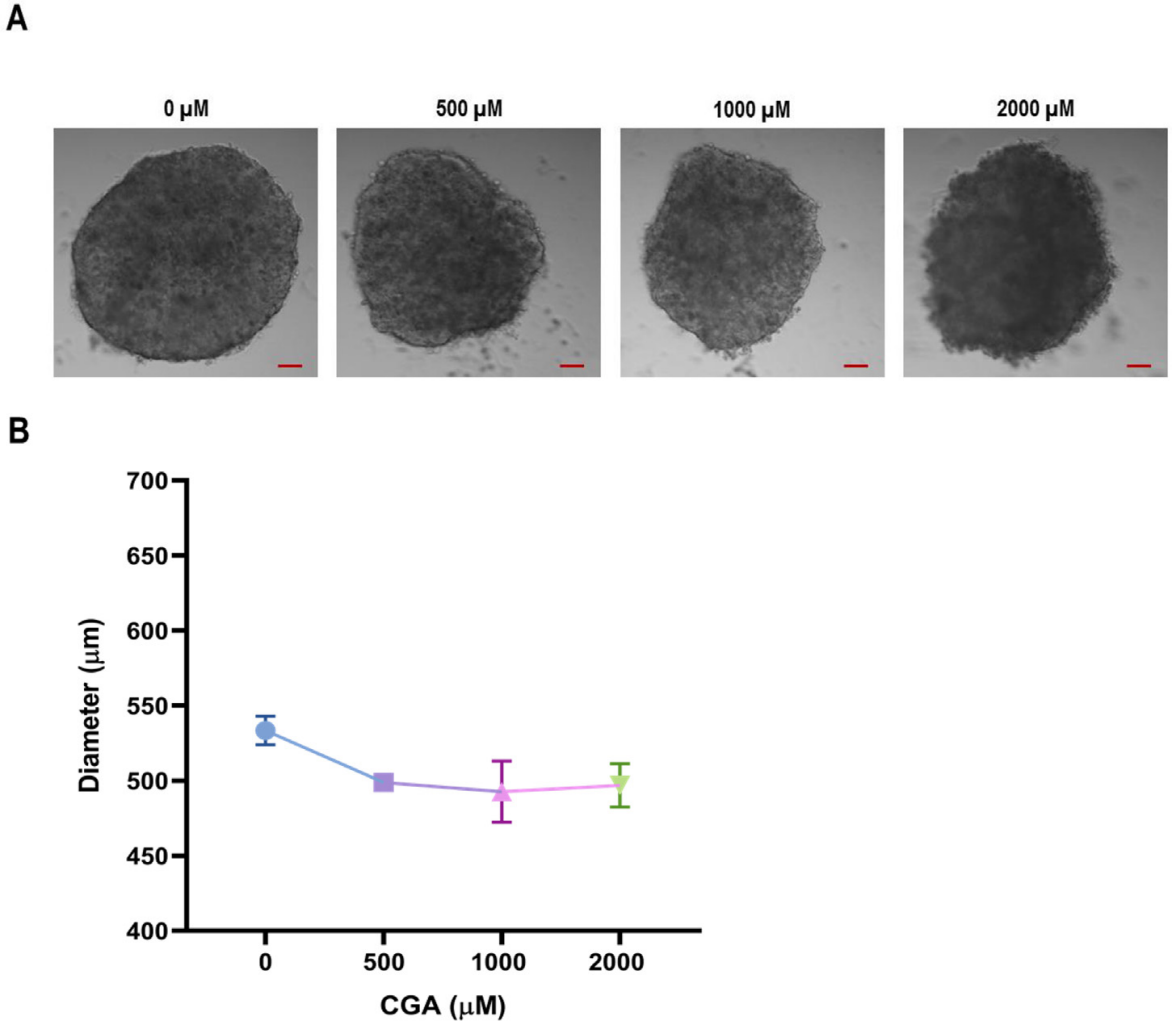
Spheroids’ average diameter after being treated with CGA. Spheroids were treated with different concentrations of CGA for 24 h and observed by light microscopy for morphological analysis on a Nikon's Eclipse Ti-S inverted microscope. (A) Representative photographs of spheroids under each treatment condition. Scale bar = 50 µm. (B) Effect of CGA on the spheroids’ diameter. The diameters were measured using ImageJ software. Data are presented as the mean ± SEM of three separate experiments, and differences were evaluated by one-way ANOVA.

Fig. 1A depicts the morphological changes in HT-29 CRC cell spheroids after treatments with CGA. Similarly, Fig. 1B shows the changes in the spheroids’ diameter due to the treatments. As observed, spheroids’ diameter started to decrease at concentrations above 500 µM.

### 2.2 Effect of Chlorogenic Acid on Cell Spheroid Viability

**Fig. 2:**
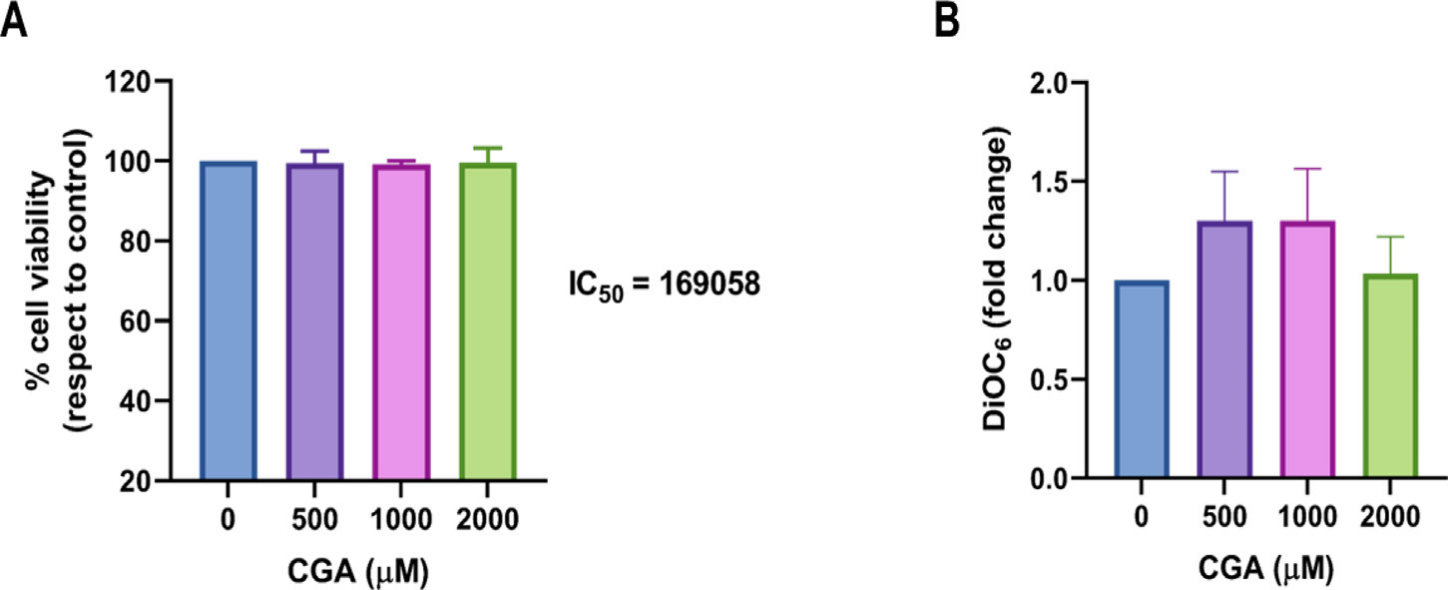
Effect of CGA on spheroid viability. Spheroids were treated with different concentrations of CGA for 24 h and then dissociated, processed, and analyzed by flow cytometry. (A) Cell viability measured by PI incorporation in response to treatment with CGA. The value next to each bar chart represents the IC50. (B) DiOC6 uptake as a measure of mitochondrial membrane polarization. Data are expressed as the mean ± SEM of three separate experiments, and differences were evaluated by one-way ANOVA.

Propidium iodide (PI) is a fluorescent dye impermeable to the cytoplasmic membrane, that is, it cannot enter cells with intact membranes. Therefore, it is a good indicator of cell viability, making it possible to distinguish between living and dead cells [13]. The percentage of viability of the HT-29 cells that formed the spheroids is shown in Fig. 2A. The concentrations analyzed in this study did not induce a cytotoxic response of the cells after 24 h of treatment with CGA. The IC_50_ value required to inhibit spheroid growth is 169058 µM.

To analyze the changes induced by CGA in the mitochondrial membrane potential, we used staining with DiOC6, a lipophilic fluorescent dye that is sensitive to mitochondria in living cells at low concentrations [14]. Fig. 2B shows that there are no significant changes under the employed conditions.

### 2.3 Effect of Chlorogenic Acid on Mitochondrial ROS of HT-29 Cell Spheroids

**Fig. 3:**
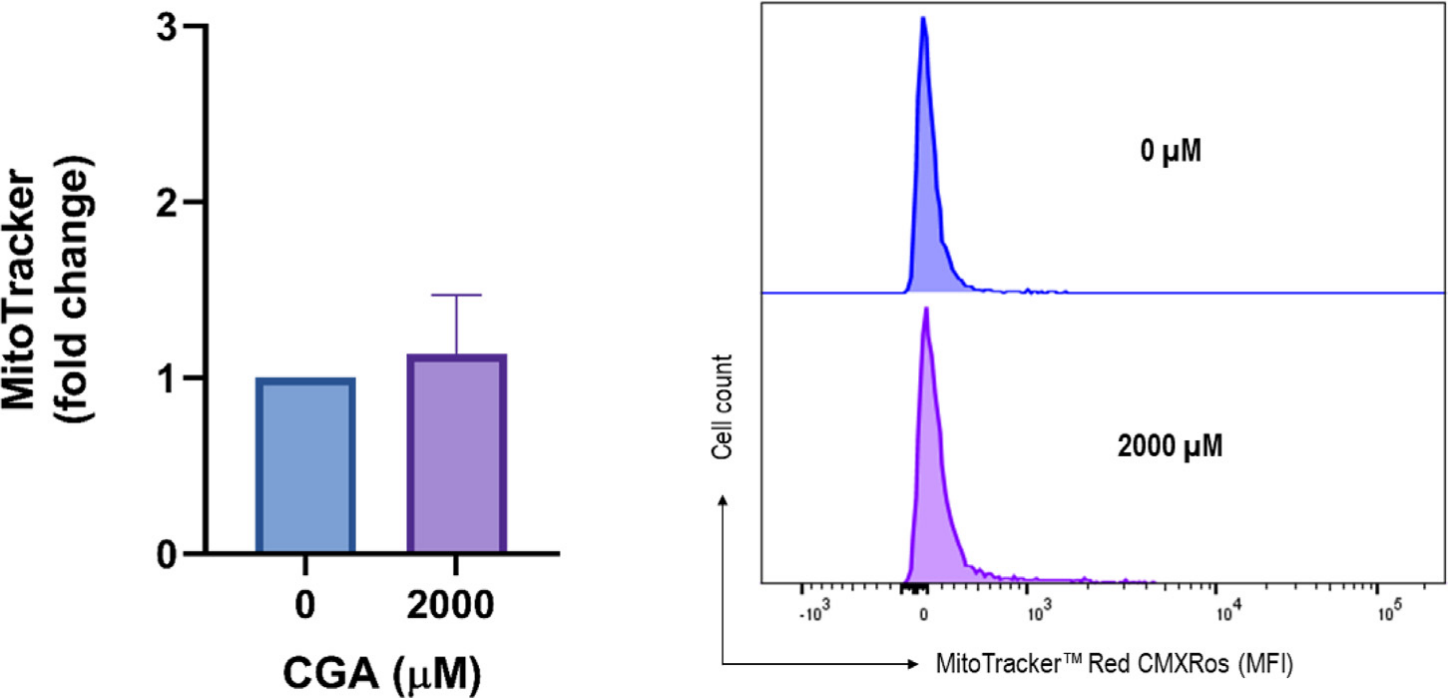
Quantification of mitochondrial ROS production in spheroids after treatment with CGA. Spheroids were treated with different concentrations of CGA for 24 h and then dissociated, processed, and analyzed by flow cytometry using MitoTracker™ Red. The bar chart shows the mean ± SEM of the MFI fold change obtained in three separate experiments. The right panel shows a representative histogram. Differences were evaluated by Student's t-test, which showed no differences between treatments and the control group.

Mitochondria are a major source of ROS in mammalian cells. ROS production contributes to mitochondrial damage in a variety of pathologies, including cancer [15,16]. Therefore, we analyzed the changes in mitochondrial ROS concentrations after treating spheroids with CGA. Fig. 3 shows a representative histogram obtained from the flow cytometry and its corresponding quantification using MitoTracker™ Red. As observed, there is a slight increase in ROS concentration in HT-29 cells evaluated at the highest treatment dose.

### 2.4 Apoptosis Detection Using Annexin V-PE/SYTOX™ Green Labeling

**Fig. 4:**
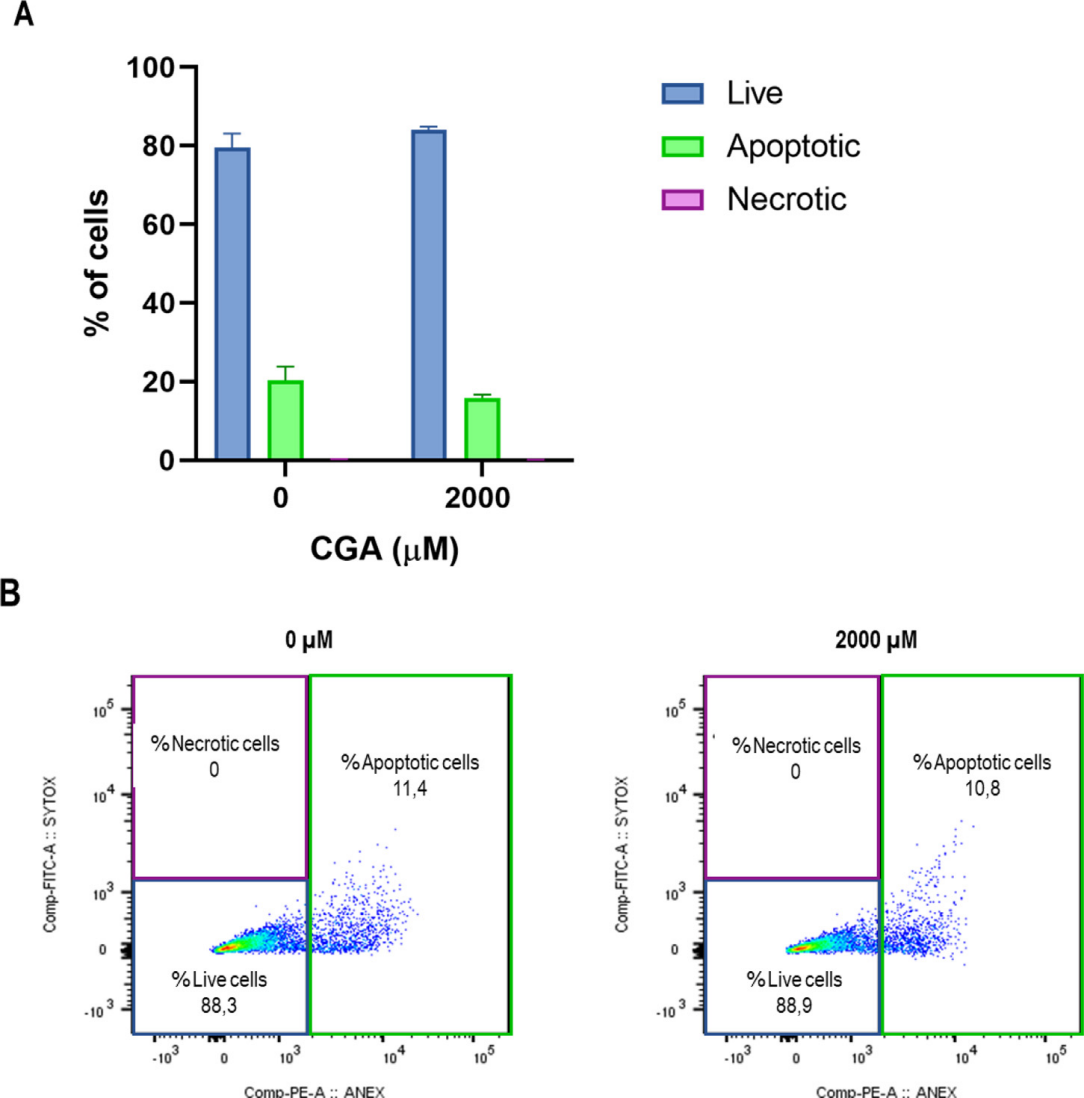
Effect of CGA on the induction of apoptosis in HT-29 cell spheroids. Spheroids were treated with different concentrations of CGA for 24 h and then dissociated, processed, and analyzed by flow cytometry using the fluorophores Annexin V-PE and SYTOX™ Green. (A) The bar chart shows the mean ± SEM of the percentage of cells in each subpopulation (positive for Annexin, positive for SYTOX™ Green, and negative for both markers). (B) A representative dot plot shows the percentage of cells in each subpopulation. The two-way ANOVA showed no difference from untreated cells.

Annexin V-PE/SYTOX™ Green co-staining of the HT-29 cells that form the spheroids and subsequent analysis by flow cytometry made it possible to differentiate the following cell populations: apoptotic cells (cells positive for Annexin), necrotic cells (cells positive for SYTOX™ Green), and viable cells (cells negative for both markers). Fig. 4 shows the quantification of HT-29 cells in each of the populations, as well as a representative dot plot for each treatment. These results indicate that the percentage of apoptotic and necrotic cells was not significantly different compared to untreated cells.

### 2.5 Cell cycle Analysis in HT-29 Cells

**Fig. 5:**
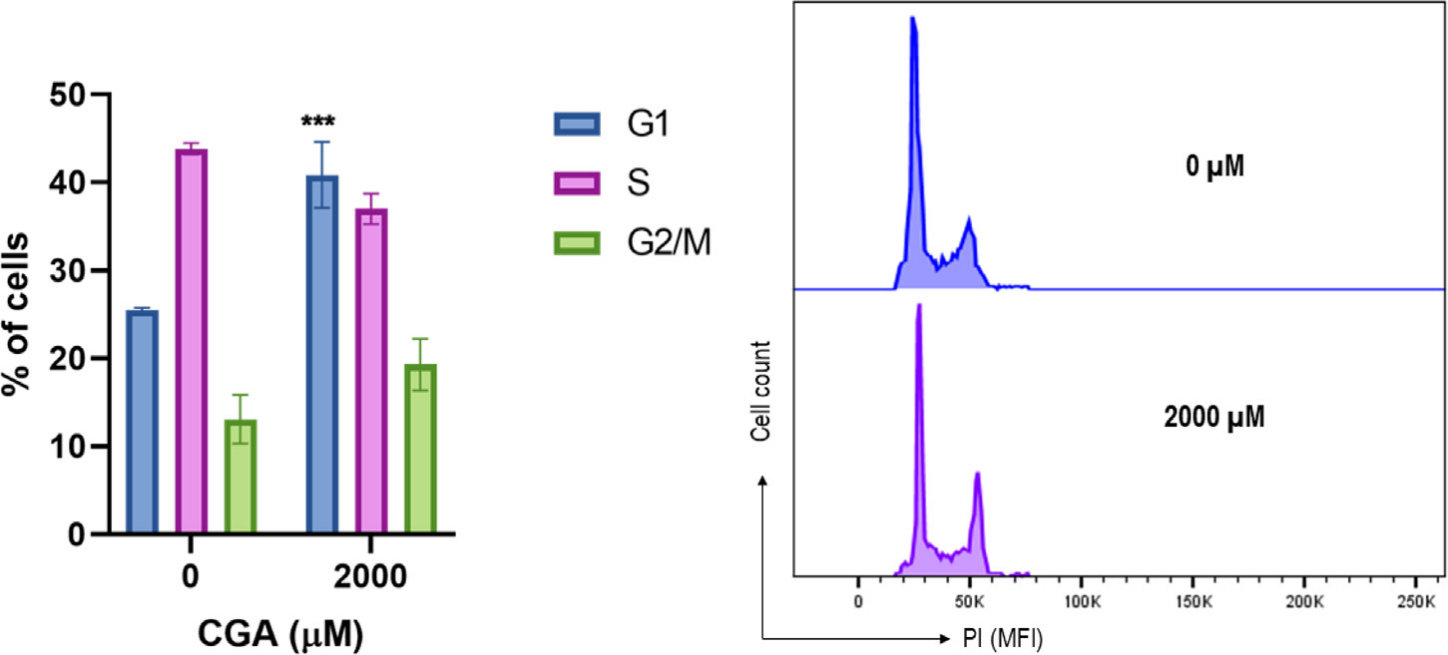
Cell cycle distribution in HT-29 cell spheroids after exposure to CGA. Spheroids were treated with different concentrations of CGA for 24 h and then dissociated, processed, and analyzed by flow cytometry using PI. The right panel shows a representative histogram of the cell cycle distribution, and the bar chart shows the mean ± SEM of the percentage of cells in the different cell cycle phases obtained in three separate experiments. The two-way ANOVA for G1, S, and G2/M populations showed differences from untreated cells, where **** *p* ≤ 0.001.

Fig. 5 shows the quantification of the HT-29 cells that formed the spheroids in each of the phases of the cell cycle. It also displays a representative histogram for each treatment, where the arrest in the G1 phase of the cell cycle is demonstrated with statistically significant data at the CGA dose evaluated (2000 µM). This arrest in the G1 phase may have been caused by DNA damage induced by the treatment [17,18].

## 3 Experimental Design, Materials and Methods

### 3.1 Preparation of Chlorogenic Acid

Prior to spheroid treatment, 3.54 mg of pure CGA (Sigma, C3878) were dissolved in 1 mL of fresh unsupplemented culture medium. The solution was vortexed until a homogeneous mixture was obtained. The final CGA concentration was 10 mM. Lastly, the necessary dilutions were made to obtain the concentrations evaluated in this study (0–2000 µM).

### 3.2 Cell Spheroid Culture

HT-29 CRC cells (ATCC®, HTB-38™) were cultured in a Roswell Park Memorial Institute (RPMI) 1640 medium supplemented with 10% fetal bovine serum (FBS) and 100 µg/mL penicillin/streptomycin. The liquid overlay technique (LOT) was employed to form the cell spheroids [19]. Then, a solution of 1% (w/v) agarose in dH_2_O was prepared and autoclaved. Subsequently, 96-well flat-bottomed plates were coated with 70 µL of liquid agarose and allowed to solidify at room temperature for 1.5 h before culturing. After this, 100 µL of a suspension containing 1 × 10^5^ cells/mL was seeded in each agarose-coated plate and centrifuged at 1000 gravities (g) for 15 min. Lastly, the cells were incubated for 72 h under standard culture conditions at 37 °C in a humidified incubator with 5% CO_2_.

### 3.3 Treatment Scheme

Once the spheroids were formed, they were treated for 24 h with various CGA concentrations using a different medium. After the treatments, the cell spheroids were dissociated by trypsinization and processed for various microscopy and flow cytometry analyses.

### 3.4 Morphological Analysis

HT-29 cells were cultured and treated under the conditions described above. For their morphological analysis, the cell spheroids were observed and photographed by light microscopy using a Nikon's Eclipse Ti-S inverted microscope.

### 3.5 Cell Viability

To measure cell viability, cytoplasmic membrane integrity and changes in mitochondrial membrane potential were evaluated using PI (Sigma, P4170) and DiOC_6_ (Molecular Probes, D273), respectively. After CGA treatments, spheroids were washed twice in phosphate-buffered saline (PBS), trypsinized, dissociated, stained with 1.5 µg/mL PI and 50 nM DiOC_6_, and incubated in the dark at room temperature for 20 min. Fluorophore incorporation after CGA treatments was quantified by flow cytometry, where 10,000 events were analyzed using a BD LSRFortessa™. Finally, the mean fluorescence intensity (MFI) was calculated using FlowJo_v10.8.1.

### 3.6 Quantification of Mitochondrial ROS

After CGA treatments, spheroids were washed twice in PBS, trypsinized, dissociated, stained with 3 µM MitoTracker™ Red (Invitrogen, M7512), and incubated at 37 °C for 30 min. Finally, dissociated cells were washed twice in PBS and 10,000 events were analyzed by flow cytometry using a BD LSRFortessa™. The MFI of MitoTracker™ Red was calculated running FlowJo_v10.8.1.

### 3.7 Detection of Necrosis and Apoptosis by Flow Cytometry

Annexin V-PE/SYTOX™ Green co-staining was performed to quantify the cell populations positive for Annexin (apoptotic cells) and positive for Sytox (necrotic cells) that formed the spheroids. After CGA treatments, spheroids were washed twice in PBS, trypsinized, dissociated, and stained for 15 min using the fluorophores and following the kit manufacturer's instructions (Molecular Probes Thermo Scientific, V35112). By flow cytometry, 10,000 events were analyzed employing a BD LSRFortessa™. Lastly, the MFI was calculated using FlowJo_v10.8.1.

### 3.8 Cell Cycle Analysis

Cell cycle progression was measured using PI as a DNA intercalating agent. After CGA treatments, spheroids were washed twice in PBS, trypsinized, dissociated, and fixed for at least 24 h using cold 70% ethanol. Finally, they were incubated with 6.7 µg/mL RNase (Sigma, R5000) and 5 µg/mL PI (Sigma, P4170) at room temperature for 30 min. By flow cytometry, 10,000 events were analyzed employing a BD LSRFortessa™. Lastly, the MFI was calculated using FlowJo_v10.8.1.

### 3.9 Statistical Analysis

GraphPad Prism V9 was employed to perform the statistical analysis and create the graphs in this paper. The data obtained represent the results from three separate experiments, one for each treatment group. Data were compared using the Student's *t*-test and ANOVA, as well as Fisher's protected least significant difference (FPLSD) tests. Data were presented as the mean ± standard error of the mean (SEM), and *p* ≤ 0.05 was considered statistically significant.

## Ethics Statement

The authors confirm that they have read and followed all the ethical requirements for publication in Data in Brief. It is important to emphasize that the current study does not involve human subjects, animal experiments, or any data collected from social media platforms. All activities, and data collection were conducted in strict accordance with ethical guidelines and principles, ensuring the integrity and responsible conduct of the research.

## CRediT authorship contribution statement

**M. Daniela Vélez:** Conceptualization, Methodology, Formal analysis, Investigation. **Johanna Pedroza-Díaz:** Conceptualization, Methodology, Investigation, Resources. **Gloria A. Santa-González:** Conceptualization, Methodology, Formal analysis, Investigation, Resources.

## Data Availability

Flow cytometry CGA datasets (Original data) (Mendeley Data).
